# Supplementary material for: Endocannabinoid 2-Arachidonoylglycerol Levels in the Anterior Cingulate Cortex, Caudate Putamen, Nucleus Accumbens, and Piriform Cortex Were Upregulated by Chronic Restraint Stress
Source: Cells. 2023 Jan 21;12(3):393. doi: 10.3390/cells12030393 (PMC9913316; doi:10.3390/cells12030393)
Supplement: Supplementary file 1 [file cells-12-00393-s001.zip › cells-2148957-supplementary.pdf]

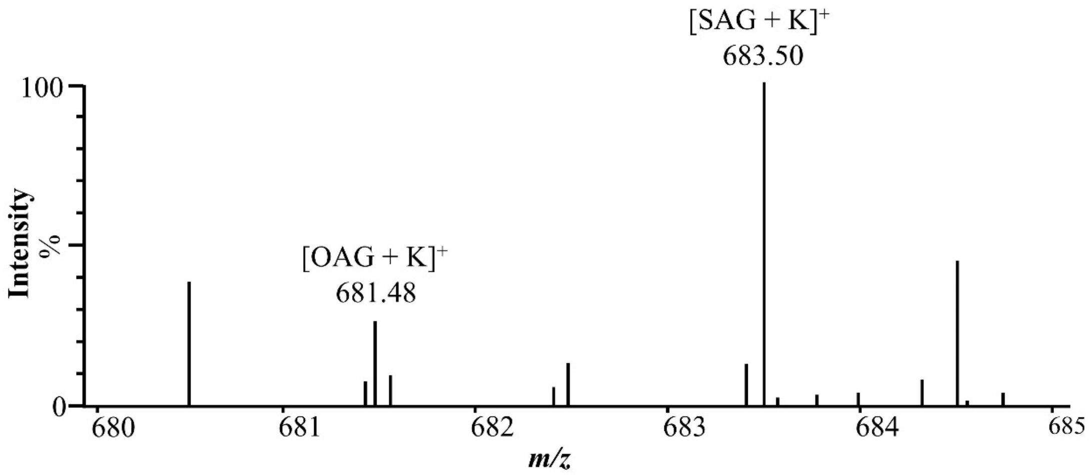

Figure S1. DESI-MSI mass spectra showed that OAG and SAG were detected in mice brain tissue by DESI-MSI in positive ion mode. OAG: 1-oleoyl-2-arachidonoyl-sn-glycerol, SAG: 1-stearoyl-2-arachidonoylglycerol.

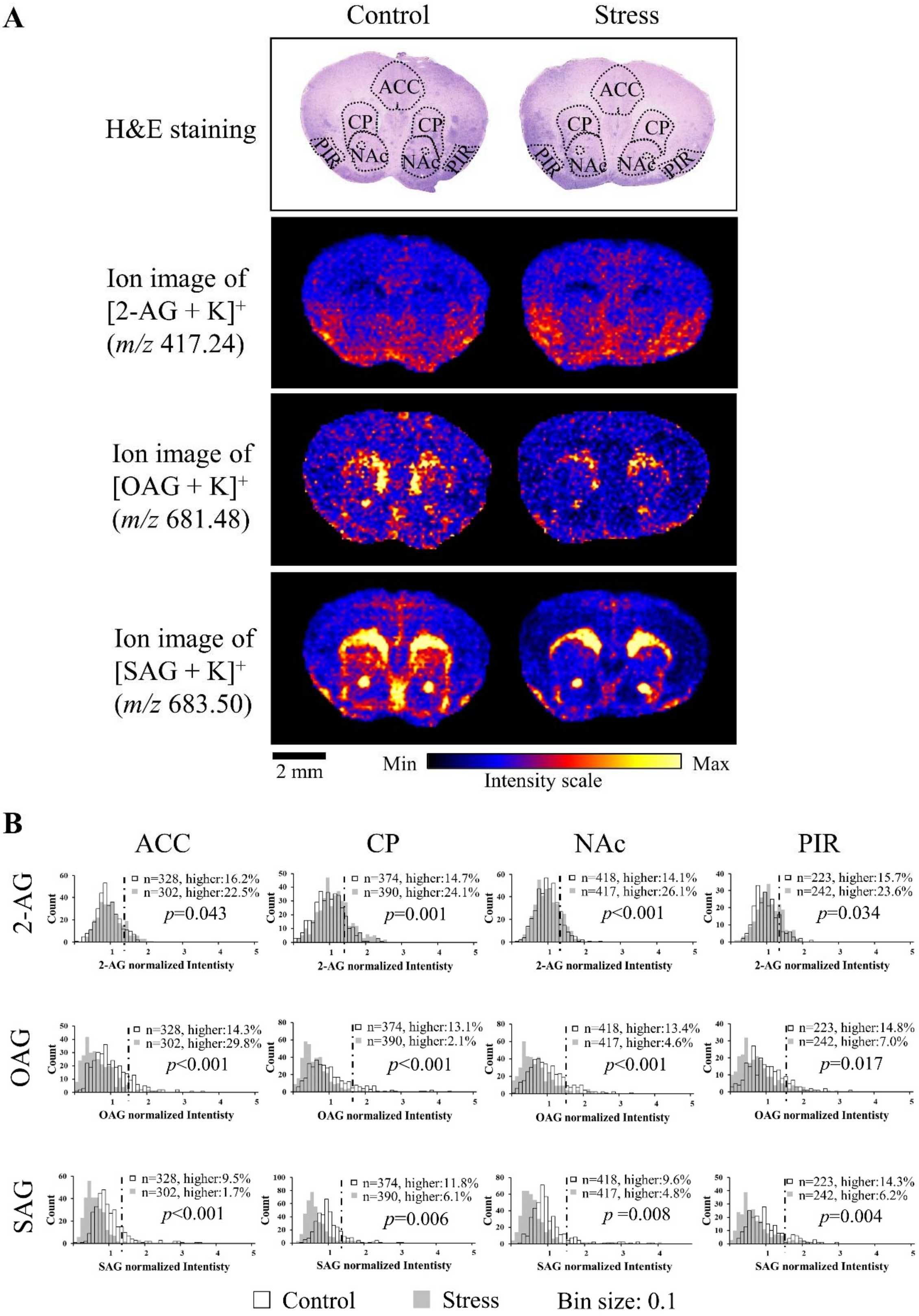

Figure S2. CRS upregulated the 2-AG levels and downregulated OAG and SAG levels in the ACC, CP, NAc, and PIR in group #2 mice. (**A**) H&E staining results and ion images of 2-AG, OAG, and SAG in coronal brain

sections of control and stressed mice (scale bar: 2 mm). ACC, CP, NAC, and PIR in control and stressed mice brains were circled by black dot lines for analysis. Anatomical annotations were referenced to Allen Brain Atlas (https://atlas.brain-map.org/). (**B**) Histograms represent the intensity change of 2-AG, OAG, and SAG in the ACC, CP, NAC, and PIR of control and stressed mice. The value in each pixel of the stress and control groups was normalized by dividing the mean of the control group. A threshold line was set as the mean + SD of the control mice. Pearson's Chi-square test was performed for lower-than-threshold and higher-than-threshold signals in these two groups (n: the number of pixels). OAG: 1-oleoyl-2-arachidonoyl-sn-glycerol, SAG: 1-stearoyl-2-arachidonoylglycerol, ACC: anterior cingulate cortex, CP: caudate putamen, NAC: nucleus accumbens, PIR: piriform cortex.

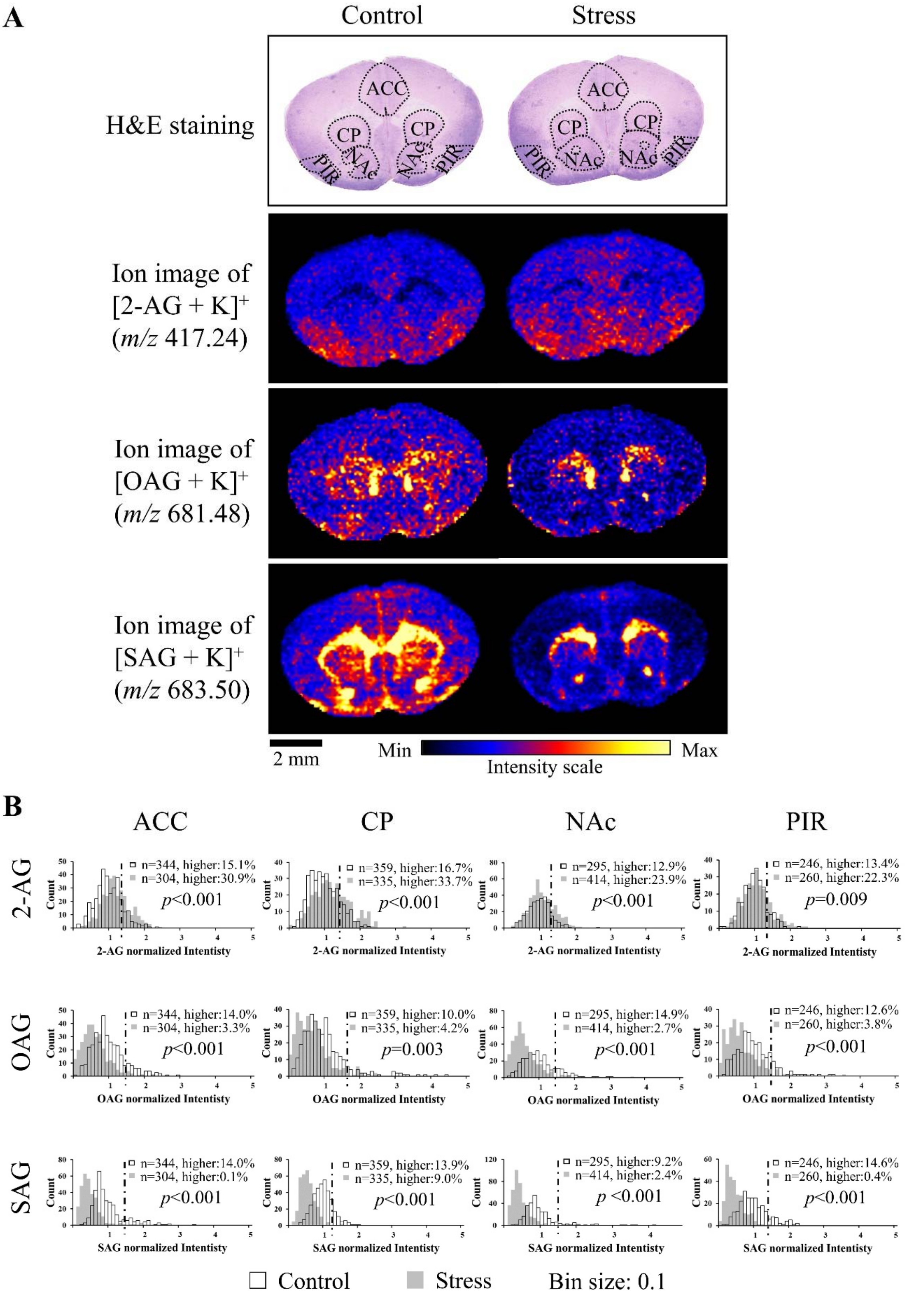

Figure S3. CRS upregulated the 2-AG levels and downregulated OAG and SAG levels in the ACC, CP, NAc, and PIR in group #3 mice. (**A**) H&E staining results and ion images of 2-AG, OAG, and SAG in coronal brain

sections of control and stressed mice (scale bar: 2 mm). ACC, CP, NAC, and PIR in control and stressed mice brains were circled by black dot line for analysis. Anatomical annotations were referenced to Allen Brain Atlas. (**B**) Histograms represent the intensity change of 2-AG, OAG, and SAG in the ACC, CP, NAC, and PIR of control and stressed mice. The meaning of the histogram, the method of analysis, and the abbreviated name are the same as in Figure S2.
